# Mixed Adenoneuroendocrine Carcinoma of the Gastroesophageal Junction: A Rare Find

**DOI:** 10.1177/2324709617750180

**Published:** 2017-12-21

**Authors:** Paurush Ambesh, Joseph Weissbrot, Sabina Ratner, Ankur Sinha, Ravikaran Patti, Jasminka Balderacchi, Michael Marcelin, Lawrence Wolf, Stephan Kamholz

**Affiliations:** 1 Maimonides Medical Center, Brooklyn, NY, USA

**Keywords:** MANEC, cancer

## Abstract

Neoplastic lesions that demonstrate neuroendocrine features are rare. However, esophageal tumors containing both adenocarcinomatous and neuroendocrine components are exceedingly rare. Mixed adenoneuroendocrine carcinomas (MANECs) are gastrointestinal tumors with both adenocarcinomatous and neuroendocrine differentiation. They have a tendency for early metastases but clinically manifest relatively late. Imaging studies are often nonspecific with regard to tumor type, and a histopathologic study of biopsy material is required for definitive diagnosis. The overall prognosis is poor. The current report describes a rare case of gastroesophageal MANEC tumor, with approximately 60% neuroendocrine and 40% adenocarcinomatous components. Since there is a dearth of concrete management guidelines for MANECs, we present possible management options to add to the existing literature.

## Introduction

Esophageal neoplastic lesions that demonstrate neuroendocrine features are exceedingly rare. Esophageal tumors containing both adenocarcinomatous and neuroendocrine components are also infrequently encountered. Mixed adenoneuroendocrine carcinomas (MANECs) are gastrointestinal tumors with both adenocarcinomatous and neuroendocrine differentiation. These tumors tend to metastasize early and clinically manifest late in the course of the illness. The overall prognosis is extremely poor. Imaging studies are often nonspecific with regard to tumor type, and histopathologic study of biopsy material is required for definitive diagnosis. The current report describes a rare case of gastroesophageal MANEC tumor, with approximately 60% neuroendocrine and 40% adenocarcinomatous components.

## Case Description

A 67-year-old man presented complaining of 3 days of dysphagia and retrosternal chest pain. The pain was described as “crushing” in nature and was constantly present, worsening when swallowing food. He described the sensation of “something is stuck in his throat” and could only swallow liquids and semisolids. He also noted chronic halitosis. There were no other specific complaints, and family history was negative for cancer. The patient had smoked 1 pack of cigarettes daily for the past 40 years.

In the emergency room, vital signs included temperature 97.9°F; heart rate 63/min; respiratory rate 20/min; blood pressure 127/75 mm Hg right arm supine.

Electrocardiogram revealed T wave inversions with ST depressions in the anterior leads. Cardiac troponin determinations (3 sets) were within normal limits, thus excluding acute cardiac ischemia.

Computed tomography of the chest and neck were performed, which demonstrated a dilated esophagus with air and soft-tissue density material.

**Figure 1.**
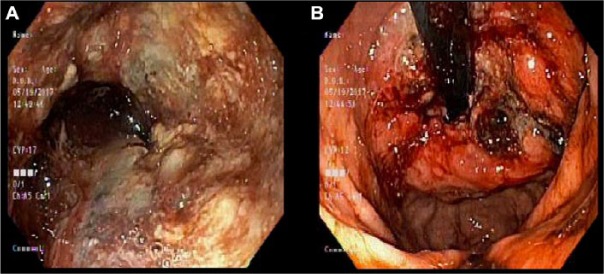
(A) Gastroesophageal junction shows large friable mass on endoscopy. (B) View from below after endoscope was passed beyond the tumor.

Upper gastrointestinal endoscopy revealed a normal appearing oropharynx. A 1-cm foreign body was noted in the distal esophagus, most likely representing a large food particle. It could not be extracted utilizing a Roth net and it passed into the stomach spontaneously. In the gastroesophageal junction, a large friable lesion was seen at 38 cm from the incisors, extending into the gastric cardia with the distal end at approximately 46 cm from the incisors. The gastroscope could be advanced beyond the mass (Figure 1).

The consistency of the lesion was hard to probing. Targeted biopsies were taken from the proximal and the distal ends of the mass. The samples were sent for pathological study.

The specimen from the proximal end of the mass demonstrated a poorly differentiated neoplasm consistent with neuroendocrine carcinoma. These was no evidence of Barrett’s esophagus. The specimen obtained from the distal aspect of the lesion revealed poorly differentiated neoplasm, most consistent with mixed adeno and neuroendocrine features. Again, there was no evidence of Barrett’s esophagus.

**Figure 2.**
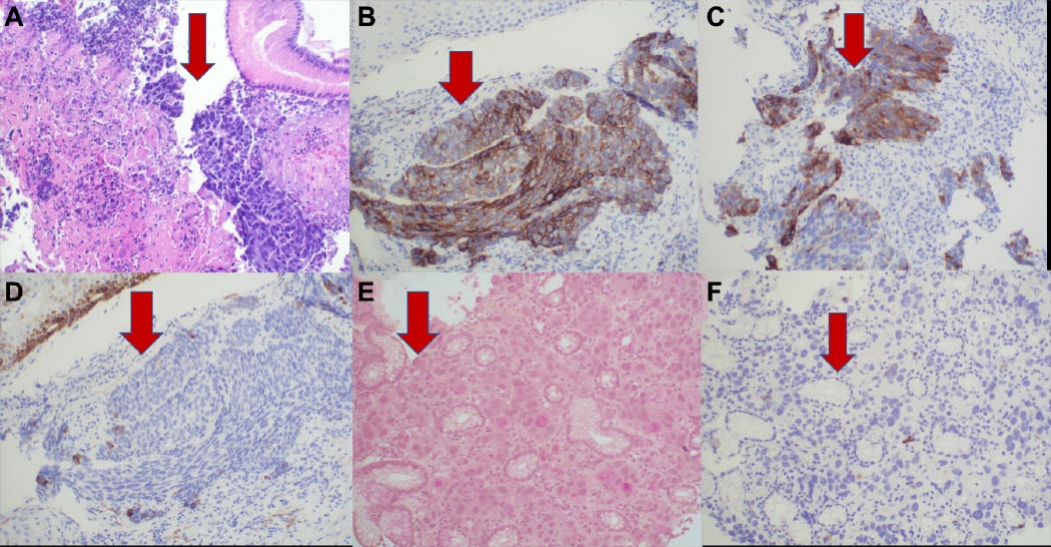
(A) Hematoxylin and eosin stain showing adenocarcinoma component. The adenocarcinoma components consist of nests of cells with scattered mucinous components. (B) Strongly positive for neural markers CD56. (C) Synaptophysin stain shows neuroendocrine component. (D) Cytokeratin stain shows adenocarcinoma component. (E) Mucicarmine stain shows mucin adenocarcinoma. (F) Synaptophysin stain shows neuroendocrine component.

Immunohistochemical stains demonstrated CDX-2 and CK labeling of pleomorphic neoplastic cells with mucicarmine, highlighting intraluminal mucin. The solid component stained with synaptophysin but did not stain with p63, CDX-2, or CK7. This staining pattern is most consistent with the rare entity known as mixed adenoneuroendocrine carcinoma of esophagus, which arises from endocrine cells in the esophagus (more commonly found in the distal esophagus; Figure 2).

Magnetic resonance imaging for cancer staging was done and revealed pT3N1M0 status of poorly differentiated carcinoma with mixed neuroendocrine/adenocarcinoma features. Distant metastases were ruled out. Our patient was evaluated by the oncology and cardiothoracic surgery teams. A joint decision to surgically excise the tumor was made. Since distant metastases was ruled out, it was decided not to administer chemotherapy. He underwent an elective tumor resection with minimally invasive Ivor-Lewis esophagogastrectomy.

He tolerated the surgery well and continues to follow-up in the oncology clinic on an outpatient basis. Our case is unique because the patient was treated by surgery alone and chemotherapy was not initiated. He is currently in remission and is doing well symptomatically.

## Discussion

The taxonomy of MANECs is confusing and meanders among amphicrine tumors, combined tumors, collision tumors, and mixed exocrine-endocrine tumors.

By definition, MANECs must contain a minimum of 30% each of adenocarcinomatous and neuroendocrine components. Three endocrine markers are used to identify the neuroendocrine component. They are chromogranin, synaptophysin, and CD56 or NSE.^1-3^

It is important to make a distinction between MANEC and collision tumors (close approximation but no admixture between neuroendocrine and adenocarcinomatous portions).

MANECs are characteristically very aggressive malignant tumors. This aggressive property is attributed to the endocrine portion of the tumor.

MANECs include a whole range of low- to high-grade lesions. The epithelial portion may demonstrate dysplasia/adenoma or invasive adenocarcinoma.

The neuroendocrine portion can be well-differentiated or poorly differentiated.

Cardier reported the first MANEC in 1924. However, the nomenclature MANEC (mixed adenoneuroendocrine carcinoma) was provided by the World Health Organization in 2010. The origin of these tumors still remains mysterious.^4-6^

MANECs possibly emanate from bidirectionally differentiated multipotent stem cells.^7^ Another hypothesis suggests that their origin is from dedifferentiated adenocarcinoma of a neuroendocrine phenotype.

Metastases appear early and prognosis is extremely poor. Metastases usually occur between regional lymph nodes and the liver. In rare instances, the peritoneum has been involved.^8^

Since MANECs are exceedingly rare, a standard treatment regimen has not yet been laid out. The National Comprehensive Cancer Network advocates cisplatin/carboplatin and etoposide for treatment.^9,10^ The median survival without therapy has been reported to be 7 to 12 months.^11^

In summary, esophageal MANEC is an extremely rare and aggressive tumor, which is discovered at the time of late-onset symptomatology. By the time it is found, metastases have already occurred. In cases where it is caught early, surgical resection might be curative. In more advanced cases where infiltration has occurred, a multidisciplinary effort among physicians, oncologists, and surgeons is warranted. Since very few cases of MANECs have been reported, there is no general consensus on treatment strategy.
